# Does retinal configuration make the head and eyes of foveate birds move?

**DOI:** 10.1038/srep38406

**Published:** 2017-01-12

**Authors:** Bret A. Moore, Luke P. Tyrrell, Diana Pita, Olaf R. P. Bininda-Emonds, Esteban Fernández-Juricic

**Affiliations:** 1 Department of Biological Sciences, Purdue University, 915 W. State Street, West Lafayette, IN 47907 USA; 2 AG Systematik und Evolutionsbiologie. IBU-Fakultät V, Carl von Ossietzky Universität Oldenburg, 26111 Oldenburg, Germany

## Abstract

Animals move their heads and eyes to compensate for movements of the body and background, search, fixate, and track objects visually. Avian saccadic head/eye movements have been shown to vary considerably between species. We tested the hypothesis that the configuration of the retina (i.e., changes in retinal ganglion cell density from the retinal periphery to the center of acute vision-fovea) would account for the inter-specific variation in avian head/eye movement behavior. We characterized retinal configuration, head movement rate, and degree of eye movement of 29 bird species with a single fovea, controlling for the effects of phylogenetic relatedness. First, we found the avian fovea is off the retinal center towards the dorso-temporal region of the retina. Second, species with a more pronounced rate of change in ganglion cell density across the retina generally showed a higher degree of eye movement and higher head movement rate likely because a smaller retinal area with relatively high visual acuity leads to greater need to move the head/eye to align this area that contains the fovea with objects of interest. Our findings have implications for anti-predator behavior, as many predator-prey interaction models assume that the sensory system of prey (and hence their behavior) varies little between species.

Animals move their eyes and heads for multiple reasons: to compensate for body movements and changes in the visual background, search for objects of interest in the environment, fixate and track moving objects, etc^1^. More specifically, in birds head and eye movements have a saccadic nature^2^,^3^,^4^. Head movements are more common than eye movements during gaze shifts in birds^5^. However, eye movements contribute more to gaze shifting during head turns of smaller compared to those of larger amplitude^6^.

The accepted view has been that eye movements are negligible or very limited in birds due to their relatively large eye size and consequently reduced mobility within the orbits^7^. However, recent studies have shown that many species of birds, including those belonging to the most diverse avian Order (Passeriformes), have quite large degrees of eye movement^8^,^9^. Additionally, the rate at which birds move their heads also varies substantially between bird species^10^. However, there has been little empirical testing at the comparative level to explain the diversity in eye and head movement behavior controlling for the effects of shared evolutionary history. Addressing this gap can help us understand the evolution of visual strategies to search for food and avoid predators, key-components of predator-prey interactions^11^.

The way the retina spatially samples the visual environment has been suggested to have a role in head and eye movements^1^. Retinal ganglion cells transfer information from the retina to the visual centers in the brain, with regions of the retina with higher ganglion cell density (i.e., centers of acute vision, such as foveae) providing more detailed spatial information about portions of the visual space than regions of the retina with lower cell density^12^. The incidence and patterns of eye and head movements vary in species with and without centers of acute vision (respectively, heterogeneous and homogenous cell density across the retina)^1^,^13^. Because the center of acute vision provides higher quality spatial information but occupies a reduced region of the retina, some motion is expected in the form of eye or head movements to align the center of acute vision to objects of interest^14^,^15^. Therefore, head and eye movements are necessary, among other tasks, to track objects with more accuracy and obtain more detailed spatial information, ultimately leading to enhanced behavioral responses (i.e., increase chances of capturing mobile prey, finding a safe spot while escaping a predator, etc.).

The avian retina is characterized by an increase in cell density from the retinal periphery to the center of acute vision^16^ (Fig. 1a–d). Interestingly, the rate of change in ganglion cell density varies between species^17^ (Fig. 1b,e). It has been hypothesized that species in which the change in cell density is more pronounced across the retina (Fig. 1a,b) would rely more on the center of acute vision to gather high quality information than species with a more gradual change in cell density (Fig. 1d,e)^17^,^18^. This hypothesis assumes that relatively high visual acuity occurs above a certain ganglion cell density threshold, which is lower than the peak cell density found in the center of acute vision (Fig. 1b,e). In species with a more pronounced rate of change in cell density, the relative size of the retinal area with high visual acuity would be smaller (Fig. 1b,c), compared to species with a more gradual change in cell density (Fig. 1e,f), leading to a greater need to move the center of acute vision through eye and/or head movements. We consequently predicted that the degree of eye movement and the head movement rate would increase in species with a more pronounced change in ganglion cell density across the retina (Fig. 1b,c) than in species with a less pronounced change (Fig. 1e,f).

The position of the center of acute vision also varies between bird species^12^,^16^, thus leading to our second prediction. If the center of acute vision is not at the center of the retina, but localized in one specific acentric region, we would expect a stronger association between head/eye movement behavior and retinal configuration in the retinal region containing the center of acute vision because the changes in ganglion cell density (and hence visual resolution) would be more prominent there than in other regions of the retina.

In this study, we tested for the first time the relationship between retinal configuration and head/eye movement behavior in 29 bird species known to have a single fovea, controlling for the effects of evolutionary history. This single-foveate retinal configuration is representative of most of the bird species studied so far^11^. Additionally, we established the average position of the center of acute vision in these single-foveate species.

## Results

The averaged Cartesian coordinates of the fovea in the 29 bird species studied were: x, −0.145 ± 0.013 (in the temporal region); and y, 0.057 ± 0.017 (in the dorsal region; Fig. 2a). The 95% confidence intervals of both coordinates did not overlap with zero: x, −0.172, −0.119; and y, 0.023, 0.092 (Fig. 2a), indicating the avian fovea is on average not at the center of the retina but it is located dorso-temporally in species with a single fovea (Fig. 2a).

The slope in cell density change differed significantly among the different retinal regions: dorsal, 3.63 ± 0.28; temporal, 3.48 ± 0.22; ventral, 3.01 ± 0.20; nasal, 2.48 ± 0.17 (F_3,84_ = 32.50, P < 0.001). The dorsal and temporal slopes did not differ significantly (P = 0.631), but they were significantly higher than the ventral and nasal slopes (P < 0.01). Consequently, the dorsal and the temporal areas of the avian retina have a more pronounced change in cell density from the periphery to the fovea. Additionally, we found that species with higher head movement rates also showed higher degree of eye movement (F_2,27_ = 3.99, P = 0.031, R^2^ = 0.13, χ = 0).

Head movement rate was positively and significantly associated with the temporal slope in cell density change (F_2,27_ = 3.39, P = 0.049, R^2^ = 0.11, χ = 0; Fig. 3a). However, head movement rate was not significantly related to the overall (F_2,27_ = 2.27, P = 0.123, R^2^ = 0.08, χ = 0), nasal (F_2,27_ = 1.34, P = 0.279, R^2^ = 0.08, χ = 0), dorsal (F_2,27_ = 1.25, P = 0.303, R^2^ = 0.04, χ = 0), and ventral (F_2,27_ = 2.77, P = 0.080, R^2^ = 0.09, χ = 0) slopes in cell density change.

We found a positive and significant association between the degree of eye movement and the overall slope in cell density across all retinal regions (F_2,27_ = 13.00, P < 0.001, R^2^ = 0.33, χ = 0). Similar significant associations were found considering each retinal region separately: nasal (F_2,27_ = 16.66, P < 0.001, R^2^ = 0.38, χ = 0; Fig. 3c), temporal (F_2,27_ = 5.43, P = 0.010, R^2^ = 0.17, χ = 0; Fig. 3c), dorsal (F_2,27_ = 12.10, P < 0.001, R^2^ = 0.31, χ = 0; Fig. 3d), and ventral (F_2,27_ = 15.46, P < 0.001, R^2^ = 0.36, χ = 0; Fig. 3e).

## Discussion

Previous studies found that the incidence of eye movements and patterns of head and eye movements are different in species with a center of acute vision present than those without one (reviewed in ref. 1). Ours is the first comparative study showing that the way the retina is configured in bird species with a single fovea (i.e., the type of center of acute vision with the highest spatial resolving power^14^) can influence head and eye movement behaviors. However, the relationships we found varied in different regions of the retina.

The avian fovea on average is placed off the retinal center. More specifically, the fovea is commonly found in the dorso-temporal region of the retina, and thus projects into the fronto-ventral region of the head (Fig. 2b,c). This suggests that the spot that provides the highest spatial visual resolution is pointing slightly below and towards the edges of the beak but without necessarily projecting into the binocular field due to the lateral placement of the orbits^19^. The fovea placement may facilitate the visual exploration of the substrate when the head is down looking for food by integrating the retinal inputs of the right and left foveae with the frontal binocular input through small amplitude lateral movements of the head^9^.

**Figure 1:**
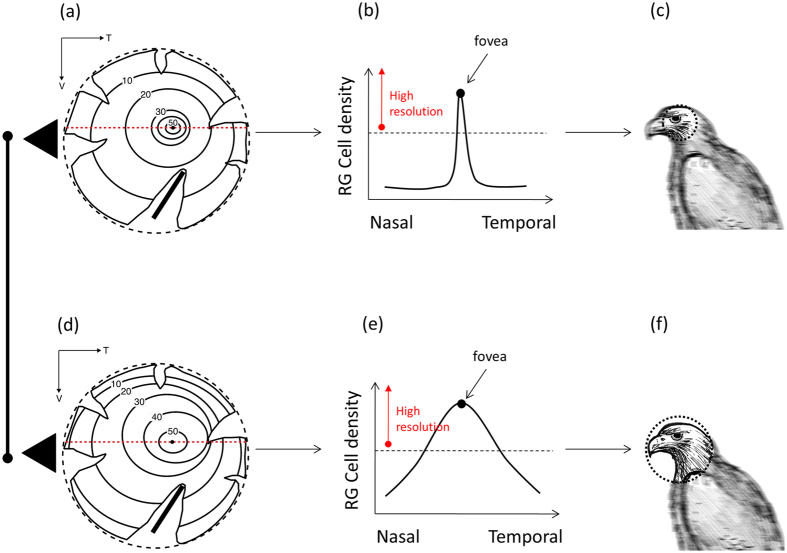
Schematic representation of the hypothesis proposed to explain the relationship between retinal configuration (variation in the density of retinal ganglion (RG) cells across the retina), the size of the area with high visual resolution in the retina, and head/eye movement behavior^17^,^18^. RG cell density increases from the retinal periphery to the fovea (i.e. center of acute vision); in this graph shown as changes in cell density from the nasal to the temporal part of the retina (**a,d**) through a sampling transect (red-dotted line). Assuming that above a threshold RG cell density animals will perceive objects with relatively high resolution, two extreme patterns of RG cell density changes can be identified: a, d (shown here as schematic representations of topographic maps with isodensity lines delimiting areas with different cell density). First, a steep change in RG cell density (**b**) will lead to a relatively smaller area of high visual resolution (**c**). Second, a smooth change in RG cell density (**e**) will lead to a relatively larger area with high visual resolution (**f**). Species with a smaller retinal area with high visual resolution (**c**) are expected to be more dependent on head/eye movements to get snapshots of a visual scene with high acuity than species with a larger retinal area with high visual resolution (**f**). Shown are the relative position of the center of acute vision (i.e., fovea, invagination of the retinal tissue) and schematic representations of a potential avian predator perceived by the species with the different types of retinal configuration. Drawing by Gabriela Sincich.

**Figure 2:**
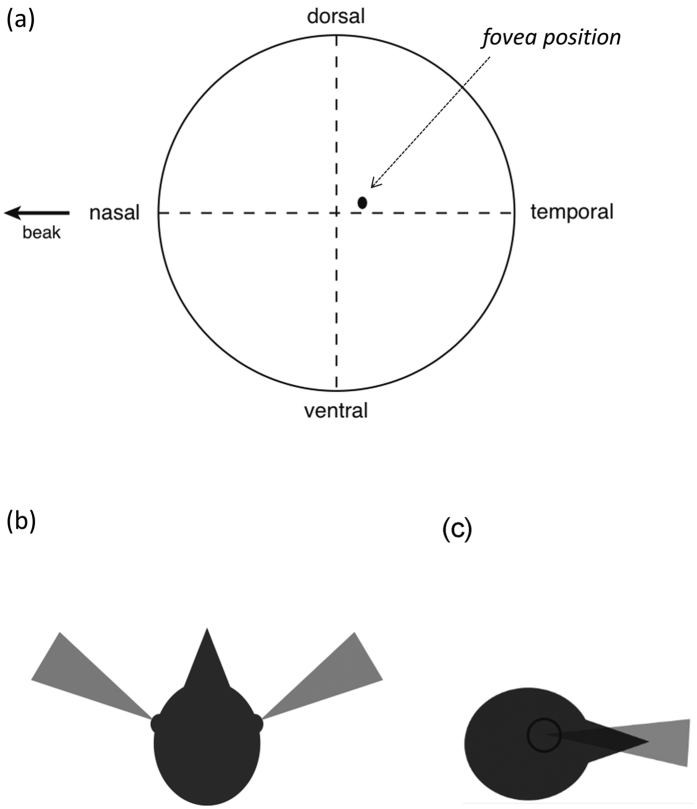
(**a**) Average position of the fovea in the 29 studied species. Top (**b**) and side (**c**) views of a bird head (black) showing the total range of fovea projections (gray) for all 29 species when the eyes were held in a resting position.

**Figure 3:**
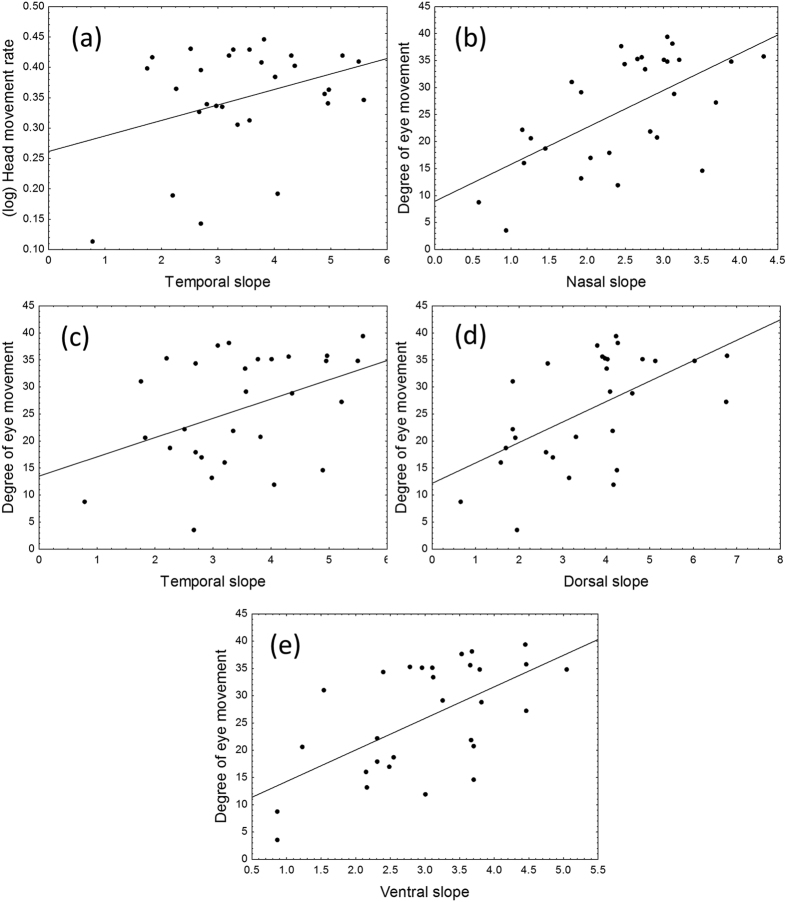
Relationships between the slope of change in ganglion cell density in different parts of the retina and head movement rate and degree of eye movement.

Our proxy of retinal configuration provided an estimate of the rate of change in spatial resolving power from the retinal periphery to the center of acute vision. In general, we found that species with a more pronounced change in spatial resolving power from the retinal periphery to the fovea tended to have higher amplitude of eye movement and higher head movement rates (but see below). Our results are consistent with the hypothesis that if the change in cell density from the periphery to the fovea is more pronounced, the retinal region above the threshold of relatively high visual acuity would be smaller and the need to move the head/eye to align this region with objects of interest would be higher compared to a more gradual change in cell density that would lead to a relatively larger retinal region above the threshold of relatively high visual acuity (Fig. 1b,e,^18^). As a result, species with higher rate of change in cell density across the retina may rely more on the center of acute vision to track objects, compensate for body and background movements, etc. This dependence on high visual acuity can translate into two mechanisms to move the center of acute vision: greater amplitude of eye movements and higher rates to move the head around, as both traits were positively associated in our studied species.

However, spatial resolving power may not be the only factor accounting for the patterns observed, as the densities of photoreceptors involved in both chromatic and achromatic vision in birds also increase from the retinal periphery to the fovea^20^. Therefore, it is also possible that chromatic and achromatic visual contrast and contrast sensitivity are also associated with the changes in spatial resolving power across different parts of the retina. This reinforces the idea that the avian fovea is more than a center of high chromatic resolution, but one also of achromatic and motion vision^20^. Overall, our findings open up the need for testing alternative retinal mechanisms driving changes in head and eye movement behavior.

Despite being significant in all retinal regions, the association between degree of eye movement and change in cell density was the weakest in the temporal region (17% compared to >30% in the other three regions). On the other hand, the association between head movement rate and change in cell density was only significant in the temporal region of the retina. We speculate that the region of the retina with the fovea may drive head movement behavior, which is more predominant in birds^5^, while the non-foveate regions of the retina may drive the different types of eye movements that to a large degree compensate for head movements^1^. This suggests that different retinal regions may play different roles in triggering eye and/or head movements, possibly influencing their orientations. Given the comparative nature of our data, measuring the orientation of head and eye movements would have been logistically challenging with the almost 30 species studied. A recent study using an avian eye tracker found that European starlings are capable of quickly moving their eyes in multiple directions (ventral, dorsal, nasal, temporal) to track a stimulus^4^. However, in the absence of a stimulus, starlings scan their visual space making spontaneous eye movements along an axis tilted downwards anteriorly and upwards posteriorly, which appears related to the visual exploration of their beaks while using their probing foraging technique^4^. Other bird species move their eyes along a horizontal axis (chicken *Gallus gallus*^21^; zebra finch *Taeniopygia guttata*^3^) or a combination of a tilted and a horizontal axes (pigeon *Columba livia*^22^). This degree of variation in the orientation of spontaneous eye movements could be related to the type of center of acute vision (fovea, area, visual streak, or combination of them) as well as the ecology of the species. The availability of new eye tracking technology^6^,^23^ could allow us to test the potential interactions between retinal morphology, head/eye movement behavior, and habitat visual complexity.

Our findings suggest that some components of anti-predator behavior (e.g., scanning) are influenced by how the avian visual system is configured. This is relevant because the predictions of many models on anti-predator behavior implicitly assume little variation in the sensory system of prey, which contradicts recent empirical evidence^9^,^11^. The implication is that some species may be at higher predation risk than others in certain habitats because of the association between retinal configuration and behavior. For instance, species with more pronounced changes in cell density across the retina may need to increase their head movement rates to a much greater extent than species with less pronounced cell density changes in visually complex habitats (e.g., wooded area) after detecting some cues that may be related to the presence of a predator (e.g., sounds of rustling leaves). Under these conditions, it is likely that the rate at which high visual resolution portions of the visual space are captured is lower in species with more pronounced changes in cell density due to their smaller retinal area with high visual acuity (Fig. 1c), potentially leading to a higher perceived risk of predation, longer flight initiation distances, and longer times at the refuge. Overall, incorporating some of the inter-specific variability in the prey visual system may help develop more realistic predictions about the interspecific variation in anti-predator behavior.

## Methods

We used 29 species that were determined to have a single fovea by cross sections of the retina (i.e., tissue invagination) or by visual inspection of wholemounted retinas (i.e., torus shape on the surface of the retina characteristic of a fovea) (Appendix 1). The species included in the study inhabit different habitats (11 in open woodlands, 6 in forests, 5 in scrubs, 3 in urban areas, 2 in grasslands, 2 in marshes), and have different diets (12 insectivores, 12 granivores, 5 omnivores) (Appendix 1). Some of the raw data on some of the species used in this study were published previously (Appendix 1), and unpublished data were obtained on species in Indiana, USA. All procedures were performed in accordance with the relevant guidelines and regulations and were approved by Purdue Animal Care and Use Committee (protocol# 09-018).

Previous publications explained in detail the methods used to collect the data for each species on degree of eye movement^24^, head movement rate^19^, and change in cell density from the retinal periphery to the fovea^16^. Here, we briefly describe these methods.

We estimated the mean degree of eye movement (°) for each species by measuring between 2 and 13 individuals per species, depending on the availability of individuals. We used a visual field apparatus with the head of the animal at the center^24^. We examined the retinal field with a Keeler Professional ophthalmoscope every 10° from below (120°) to above (60°) the bill (i.e., coordinate system where 0° was directly above the head and 90° was in front of the head). We measured the degree (amplitude) of eye movement at each elevation by encouraging the animal to converge and diverge maximally its eyes using sounds. The difference between the converged and diverged eye positions was considered the degree of eye movement.

We estimated head movement rates (number of changes in any kind of head position while head-up per min) from videos by measuring between 9 and 12 individuals per species (for a similar approach see refs 10, 19 and 25). We obtained videos from two sources: the Macaulay Library (http://macaulaylibrary.org) and our own recordings. We recorded individuals in rural and suburban areas in Tippecanoe County (IN, USA) using a JVC Everio camcorder between 0700 and 1700 hrs. We minimized the chances of re-sampling the same individual by following the individual that had been recorded for a few minutes and moving in the opposite direction at least 50 m before finding a new individual. We recorded individuals showing scanning and foraging behavior, but avoided individuals that were singing, flying, or interacting with conspecifics. We measured the number of changes in head position (lateral, up, down, etc.) while the animal was in a head-up body posture per min (head-movement rate). We did not discriminate between different types of head movements or their amplitude because videos were recorded from different angles.

We extracted, wholemounted, and stained retinas for retinal ganglion cells following the procedures presented in ref. 26. After euthanasia, the eyes were removed and axial length was measured with calipers (0.01 mm accuracy). We then hemisected each eye at the ora serrata, removed vitreous humor using forceps and spring scissors, and placed the eyecup in 4% paraformaldehyde. After fixation, we processed the retina following the procedures in refs 10 and 26. Ganglion cells were stained with cresyl violet. We used an Olympus BX51 microscope at 1000x and an Olympus S97809 camera to capture images at different sites across each retina using Stereo Investigator (MBF Bioscience, Williston, Vermont). Ganglion cells were then counted to estimate density (cells/sq.mm) in 50 mm × 50 mm counting frames in each of the sites using ImageJ (http://imagej.nih.gov/ij/). Other cell types within the ganglion cell layer (e.g. amacrine, glial cells, etc.) were selectively excluded from our counts by morphological identification^27^,^28^,^29^. We built topographic maps showing the variation in ganglion cell distribution across the retina following ref. 26.

Using the topographic maps, we measured the position of the fovea with a Cartesian coordinate system (x, y) relative to the center of the retina (method detailed in ref. 16). Positive x-values indicate temporal and negative x-values indicate nasal, and positive y-values indicate dorsal and negative y-values indicate ventral positions of the fovea. We measured gradients in ganglion cell density on the topographic maps by establishing transects across the nasal, temporal, dorsal, and ventral retinal axes, centered on the fovea^16^. Sampling points were laid out and the averaged cell density recorded. We plotted these cell density values in each axis and then fitted a trend line. The slope of this line was used as the proxy of the change in cell density from the retinal periphery to the fovea (details in ref. 16), with higher and lower slope values indicating a more and less pronounced cell density change, respectively. We estimated the averaged nasal, temporal, ventral, dorsal, and overall (all four axes) slopes based on 2 to 5 topographic maps per species. This proxy was independent of the size of the retina (which is positively associated with body size^30^), as the relationship between (log) eye axial length and the averaged slope across all axes was not significant (F_2,27_ = 0.43, P = 0.658, R^2^ = −0.02, χ = 1), controlling for phylogenetic relatedness (see below).

### Statistical analysis

Head movement rate was log transformed to meet model assumptions and we present means ± standard errors throughout. We first established the differences in nasal, temporal, ventral, and dorsal slopes using a general linear model and conducted pairwise comparisons with Tukey-tests. To assess the relationship between retinal configuration and eye and head movement behaviors, we conducted phylogenetic generalized least squares models (PGLS^31^) to account for the effects of shared evolutionary history of these species using a purpose-built phylogeny based on a maximum likelihood analysis of DNA sequence data (see below). We ran the PGLS analyses using the Caper package^32^ in R^33^. Each of the 29 species was represented by a single averaged data point in our dataset. We corroborated that our results met the model assumptions by visually inspecting the distribution of residuals and the fitted vs. the residual values. We also checked for outliers (samples with values >3 or <−3) but did not to detect any.

To control for potential similarities arising from phylogenetic relatedness (and not selection pressure), we constructed an evolutionary tree for our focal species using DNA sequence data from GenBank. A multigene data set (seven mtDNA genes plus 1 nDNA gene; see Appendix 2) was purpose-built for the analysis by downloading all sequence data for the focal species (from GenBank release number 182) and mining homologous genes using the Perl Script GenBankStrip.pl v2.1. Thereafter, for each gene, the individual sequences were aligned to one another using MUSCLE v3.8.31^34^ for non-coding genes and MUSCLE as driven by the Perl script transAlign v1.3^35^ for protein-coding genes (all Perl scripts can be downloaded from http://www.uni-oldenburg.de/ibu/systematik-evolutionsbiologie/programme/). All automated alignments were then corrected afterwards by eye as needed and pruned down to the set of focal taxa using the Perl script seqCleaner.pl v1.2. The Mississippi Alligator, *Alligator mississippiensis*, was also added as the outgroup species to define the root of the phylogenetic tree. Phylogenetic analysis of the combined data set (6661 bp for eight genes; accession numbers provided in Appendix 2) was performed in a maximum likelihood (ML) framework using the program RAxML v7.2.8^36^. A combined rapid bootstrap algorithm (with 1000 replicates) and ML search^37^ was used to derive the final tree, with the optimal model of evolution being fitted to each individual gene partition.

## Additional Information

**How to cite this article**: Moore, B. A. *et al*. Does retinal configuration make the head and eyes of foveate birds move?. *Sci. Rep.* **7**, 38406; doi: 10.1038/srep38406 (2017).

**Publisher's note:** Springer Nature remains neutral with regard to jurisdictional claims in published maps and institutional affiliations.

## Supplementary Material

Supplementary Appendix 1

Supplementary Information 2
