# Supplementary material for: Does retinal configuration make the head and eyes of foveate birds move?
Source: Sci Rep. 2017 Jan 12;7:38406. doi: 10.1038/srep38406 (PMC5228126; doi:10.1038/srep38406)
Supplement: Supplementary Appendix 1 [file srep38406-s1.pdf]

Appendix 1

Manuscript title: Does retinal configuration make the head and eyes of foveate birds move?

Authors: Bret A. Moore, Luke P. Tyrrell, Diana Pita, Olaf R.P. Bininda-Emonds & Esteban Fernández-Juricic

Appendix 1: Sources of data for the 29 species of birds with a single fovea included in this study. Habitat and diet data were obtained from https://www.allaboutbirds.org/

Family	Genus Species	Common Name	Head movement rate	Degree of eye movement	Retinal configuration	Habitat	Diet
Anatidae	*Branta canadensis*	Canada Goose	Unpublish. data	Fernandez-Juricic et al. 2011b	Fernandez-Juricic et al. 2011b	Marsh	Seeds
Cardinalidae	*Cardinalis cardinalis*	Northern Cardinal	Unpublish. data	Unpublish. data	Unpublish. data	Open woodland	Seeds
Cardinalidae	*Passerina cyanea*	Indigo Bunting	Unpublish. data	Unpublish. data	Unpublish. data	Open woodland	Insects
Columbidae	*Zenaida macroura*	Mourning Dove	Unpublish. data	Blackwell et al. 2009	Dolan & Fernandez-Juricic 2010	Open woodland	Seeds
Corvidae	*Cyanocitta cristata*	Blue Jay	Unpublish. data	Unpublish. data	Unpublish. data	Forest	Omnivore
Emberizidae	*Junco hyemalis*	Dark-eyed Junco	Moore et al. 2015	Moore et al. 2015	Moore et al. 2015	Forest	Seeds
Emberizidae	*Melospiza melodia*	Song Sparrow	Moore et al. 2015	Moore et al. 2015	Moore et al. 2015	Open woodland	Insects
Emberizidae	*Pipilo crissalis*	California Towhee	Unpublish. data	Fernandez-Juricic et al. 2011a	Fernandez-Juricic et al. 2011a	Scrub	Seeds
Emberizidae	*Pipilo crythrophthalmus*	Eastern Towhee	Moore et al. 2015	Moore et al. 2015	Moore et al. 2015	Scrub	Omnivore
Emberizidae	*Spizella arborea*	American Tree Sparrow	Moore et al. 2015	Moore et al. 2015	Moore et al. 2015	Open woodland	Seeds
Emberizidae	*Spizella passerina*	Chipping sparrow	Moore et al. 2015	Moore et al. 2015	Moore et al. 2015	Open woodland	Seeds
Emberizidae	*Spizella pusilla*	Field Sparrow	Moore et al. 2015	Moore et al. 2015	Moore et al. 2015	Scrub	Insects
Emberizidae	*Zonotrichia albicollis*	White-throated Sparrow	Moore et al. 2015	Moore et al. 2015	Moore et al. 2015	Forest	Seeds
Emberizidae	*Zonotrichia leucophrys*	White-crowned	Unpublish. data	Fernandez-Juricic et al. 2011a	Fernandez-Juricic et al. 2011a	Scrub	Insects

		Sparrow					
Fringillidae	*Carduelis tristis*	American Goldfinch	Baumhardt et al. 2014	Baumhardt et al. 2014	Baumhardt et al. 2014	Open woodland	Seeds
Fringillidae	*Carpodacus mexicanus*	House Finch	Unpublish. data	Fernandez-Juricic et al. 2008	Dolan & Fernandez-Juricic 2010	Town	Seeds
Icteridae	*Agelaius phoeniceus*	Red-winged Blackbird	Unpublish. data	Unpublish. data	Unpublish. data	Marsh	Insects
Icteridae	*Molothrus ater*	Brown-headed Cowbird	Unpublish. data	Blackwell et al. 2009	Dolan & Fernandez-Juricic 2010	Grassland	Seeds
Icteridae	*Quiscalus quiscula*	Common Grackle	Unpublish. data	Unpublish. data	Unpublish. data	Open woodland	Omnivore
Icteridae	*Sturnella magna*	Eastern Meadowlark	Tyrrell et al. 2013	Tyrrell et al. 2013	Tyrrell et al. 2013	Grassland	Insects
Mimidae	*Dumetella carolinensis*	Gray Catbird	Unpublish. data	Unpublish. data	Unpublish. data	Open woodland	Insects
Mimidae	*Toxostoma rufum*	Brown Thrasher	Unpublish. data	Unpublish. data	Unpublish. data	Scrub	Omnivore
Paridae	*Baeolophus bicolor*	Tufted Titmouse	Moore et al. 2013	Moore et al. 2013	Moore et al. 2013	Forest	Insects
Paridae	*Poecile atricapilla*	Carolina Chickadee	Moore et al. 2013	Moore et al. 2013	Moore et al. 2013	Forest	Omnivore
Passeridae	*Passer domesticus*	House Sparrow	Unpublish. data	Fernandez-Juricic et al. 2008	Dolan & Fernandez-Juricic 2010	Town	Seeds
Sittidae	*Sitta carolinensis*	White-breasted Nuthatch	Moore et al. 2013	Moore et al. 2013	Moore et al. 2013	Forest	Insects
Sturnidae	*Sturnus vulgaris*	European starling	Unpublish. data	Unpublish. data	Dolan & Fernandez-Juricic 2010	Town	Insects
Troglodytidae	*Troglodytes aedon*	House Wren	Unpublish. data	Unpublish. data	Unpublish. data	Open woodland	Insects
Turdidae	*Turdus migratorius*	American Robin	Unpublish. data	Unpublish. data	Unpublish. data	Open woodland	Insects

References

Baumhardt P.E., B.A. Moore, M. Doppler & E. Fernández-Juricic. 2014. Do American goldfinches see their world like passive prey foragers? A study on visual fields, retinal

topography, and sensitivity of photoreceptors. Brain, Behavior and Evolution 83: 181-198.

Blackwell, B.F, E. Fernández-Juricic, T.W. Seamans & T. Dolan. 2009. Avian visual system configuration and behavioural response to object approach. Animal Behavour 77: 673-684.

Dolan, T. & E. Fernández-Juricic. 2010. Retinal ganglion cell topography of five species of ground foraging birds. Brain, Behavior and Evolution 75: 111-121.

Fernández-Juricic, E. M.D. Gall, T. Dolan, V. Tisdale & G.R. Martin. 2008. The visual fields of two ground foraging birds, House Finches and House Sparrows, allow for simultaneous foraging and anti-predator vigilance. Ibis 150: 779-787.

Fernández-Juricic, E., M.D. Gall, T. Dolan, C. O'Rourke, S. Thomas, J.R. Lynch. 2011a. Visual systems and vigilance behaviour of two ground-foraging avian prey species: white-crowned sparrows and California towhees. Animal Behaviour 81: 705-713.

Fernández-Juricic, E. B. Moore, M. Doppler, J. Freeman, B.F. Blackwell, S.L. Lima & T.L. DeVault. 2011b. Testing the terrain hypothesis: Canada geese see their world laterally and obliquely. Brain, Behavior and Evolution 77: 147-158.

Moore B.A., M. Doppler, J.E. Young & E. Fernández-Juricic. 2013. Interspecific differences in the visual system and scanning behavior of three forest passerines that form heterospecific flocks. Journal of Comparative Physiology A 199: 263–277.

Moore, B.A., D. Pita, L.P. Tyrrell & E. Fernández-Juricic. 2015. Vision in avian emberizid foragers: maximizing both binocular vision and fronto-lateral visual acuity. Journal of Experimental Biology 218: 1347-1358.

Tyrrell, L.P., B.A. Moore, C. Loftis & E. Fernández-Juricic. 2013. Looking above the prairie: localized and upward acute vision in a native grassland bird. Scientific Reports 3, Article number: 3231.
