# Supplementary material for: Does retinal configuration make the head and eyes of foveate birds move?
Source: Sci Rep. 2017 Jan 12;7:38406. doi: 10.1038/srep38406 (PMC5228126; doi:10.1038/srep38406)

Appendix 2

Manuscript title: Does retinal configuration make the head and eyes of foveate birds move?

Authors: Bret A. Moore, Luke P. Tyrrell, Diana Pita, Olaf R.P. Bininda-Emonds & Esteban Fernández-Juricic

Appendix 2: Maximum-likelihood phylogeny of the 29 focal bird species examined in this study using DNA sequence data obtained from GenBank. The tree was rooted using the Mississippi Alligator (*Alligator mississippiensis*; not shown) and branch lengths are proportional to the average number of substitutions per site per unit time. Bootstrap values above the nodes indicate the degree of support for the indicated relationship from 0 (none) to 100 (full).

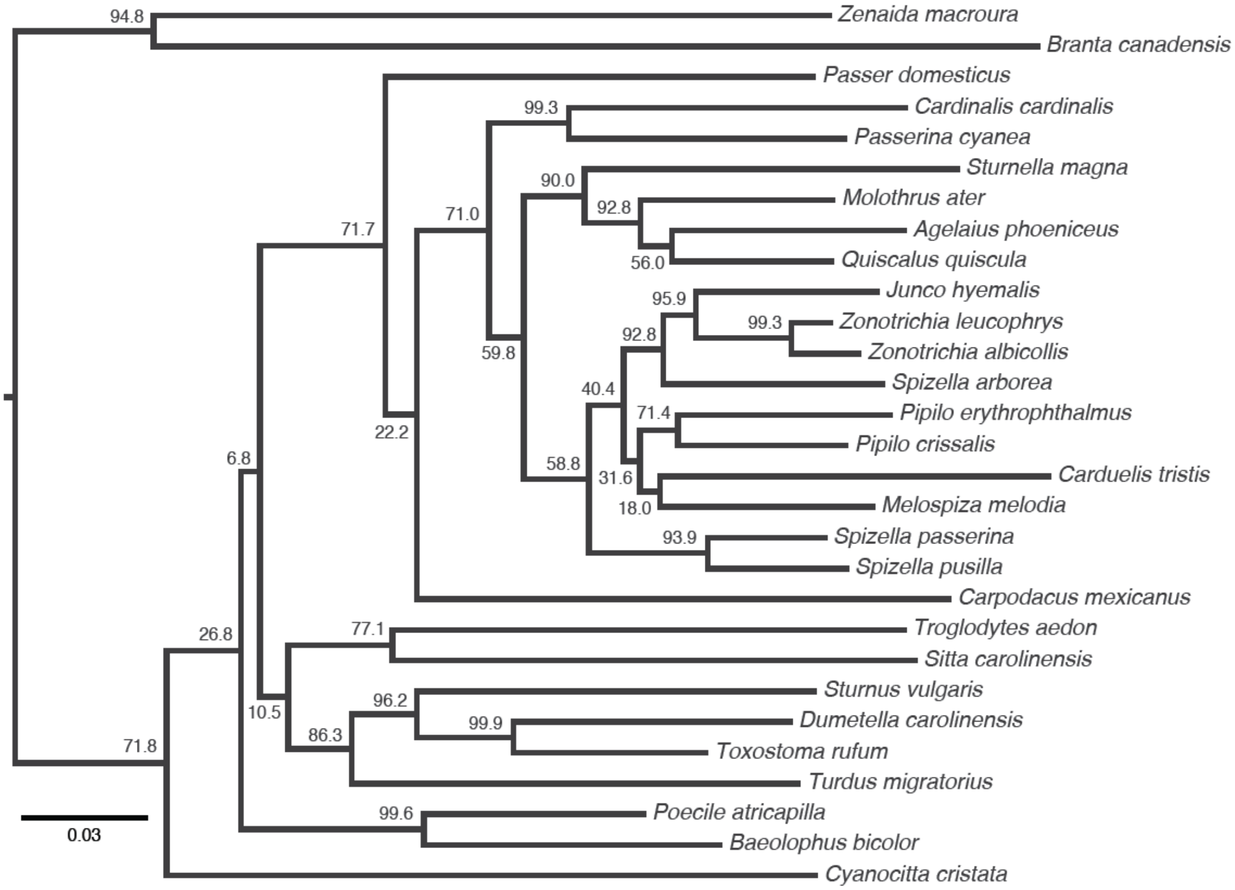

Supplement: Supplementary Information 2 [file srep38406-s2.pdf]
